# The diffuse-type gastric cancer epidemiology enigma

**DOI:** 10.1186/s12876-020-01354-4

**Published:** 2020-07-13

**Authors:** Paulo Pimentel Assumpção, Williams Fernandes Barra, Geraldo Ishak, Luiz Gonzaga Vaz Coelho, Felipe José Fernandez Coimbra, Helano Carioca Freitas, Emmanuel Dias-Neto, M. Constanza Camargo, Moyses Szklo

**Affiliations:** 1 Núcleo de Pesquisas em Oncologia, Universidade Federal do Pará, Belém, Pará Brazil; 2 Serviço de Cirurgia, Hospital Universitário João de Barros Barreto, Universidade Federal do Pará, Belém, Pará Brazil; 3 Universidade Federal de Minas Gerais, Belo Horizonte, Minas Gerais Brazil; 4 Department of Abdominal Surgery, A.C.Camargo Cancer Center, São Paulo, SP Brazil; 5 Departament of Clinical Oncology, A.C.Camargo Cancer Center, São Paulo, Brazil; 6 Medical Genomics Laboratory, CIPE/A.C.Camargo Cancer Center, São Paulo, Brazil; 7 Division of Cancer Epidemiology and Genetics, National Cancer Institute, National Institutes of Health, Rockville, MD USA; 8 Department of Epidemiology, Johns Hopkins Bloomberg School of Public Health, Baltimore, MD USA

## Abstract

**Background:**

Intestinal and diffuse gastric adenocarcinomas differ in clinical, epidemiological and molecular features. However, most of the concepts related to the intestinal-type are translated to gastric adenocarcinoma in general; thus, the peculiarities of the diffuse-type are underappreciated.

**Results:**

Besides its growing importance, there are many gaps about the diffuse-type carcinogenesis and, as a result, its epidemiologic and pathogenetic features remain poorly understood.

**Conclusions:**

Alternative hypotheses to explain these features are discussed, including the role of the gastric microbiota, medical therapies, and modifications in the stomach’s microenvironment.

## Background

Gastric adenocarcinoma (GAC) is a leading cause of cancer-related deaths, although a reduction in incidence has been observed [1, 2]. Whereas these are different histology entities [3], intestinal and diffuse Lauren’s GAC types are jointly considered when interpreting epidemiological data, and when contemplating interventional approaches aiming to reduce gastric cancer (GC) burden [1, 2]. Since the intestinal-GAC has been historically the most often studied type, data from this tumor are usually extrapolated to the diffuse-GAC. However, when examining and interpreting carcinogens and the epidemiology of diffuse-type carcinogenesis, inconsistences with regard to intestinal-type are evident [3]. These differences should be considered when trying to understand its specific pathogenesis and to apply this knowledge to develop interventional approaches. Our purpose in this commentary is to discuss some of the issues surrounding the diffuse-type GAC and to offer hypothesis to shed light on these features.

### Epidemiology

GC was responsible for more than 1,000,000 new cases in 2018 with an estimated 783,000 deaths, being the fifth most frequent and the third leading cause of cancer death. The global incidence of GC varies markedly, with the highest burden observed in some of the less developed areas of the world. Asia, Eastern Europe and South America account for most GC cases and, thus, for their high mortality rates [1]. GAC is the most common type of GC, being classically classified, according to Lauren, as intestinal- or diffuse-types [2].

GAC incidence has been declining since 1930’s [1], mainly due to the marked decrease of the intestinal-type. A decreasing trend of the intestinal- and a stable or increasing trend of the diffuse-type have been found in both high and low GC risk areas [4, 5]. However, the incidence in Korean Americans has declined during recent years, for both cardia and non-cardia sites and for both intestinal- and diffuse-type histology [6]. Several factors can explain the decreased incidence of GAC, including improvement of food preservation following the widespread use electricity and refrigeration replacing the consumption of salt-preserved foods [1, 4, 5] and, most importantly, the decrease in *H. pylori* infection rates, possibly resulting from better hygiene habits and the broad use of antibiotics [7]. However, the effects of these factors over the incidence of the diffuse-type appear to be flimsy. Indeed, risk of diffuse GAC has been generally attributed to a weaker influence of environmental factors and a stronger relevance of genetic factors [3]. Lauren’s intestinal and diffuse histological types have different prognoses. The diffuse-type generally presents reduced survival when compared to the intestinal-type [8].

### Etiology

The knowledge of environmental factors behind GAC has fundamental implications for primary prevention. Whereas case-control studies suggest an inverse association of fruit and vegetables consumption with the risk of GAC in different populations [9], data from prospective studies are less consistent with regard to the protective role of diet [10]. Additionally, the genetic factors attributed to the diffuse-type non-familial GAC remain elusive.

*H. pylori* infection is recognized by World Health Organization as a necessary cause of non-cardia gastric cancer, irrespective of Lauren’s subtype classification [7]. Nevertheless eradication of *H. pylori* seems to reduce the risk of gastric cancer [11], including the decrease in incidence of metachronous tumors among patients that were effectively treated after endoscopic resection of early gastric cancer [12] and also among patients with a family history of gastric cancer [13], there are controversial reports about this theme [14], in special, regarding the risk for diffuse type GC. Take et al (2020) demonstrated an increased risk for diffuse type gastric cancer 10 years after *H. pylori* eradication [15], showing the necessity of maintaining endoscopic follow-up for many years after effective *H. pylori* eradication.

Considering that *H. pylori* prevalence has been reduced both by lower exposure to, and higher level of bacteria eradication, a reduction of both intestinal- and diffuse-types would have been expected. However, the reduction of intestinal-type GAC incidence has not been followed by a parallel decrease of the diffuse-type [4, 5], which suggests that either *H. pylori* or other risk factors for the intestinal-type are not as strongly related to the diffuse-type [3–5], or that other risk factors to which the latter type is attributed has increased over time, thus, offsetting the decrease of *H. pylori* prevalence. These data strengths the hypothesis about the considered unexplained peculiarities involving diffuse type GC, including the etiological role of *H. pylori*, in diffuse tumors.

### Age of occurrence and gender differences

The diffuse GAC seems to occur at an earlier age than the intestinal GAC [3, 4]. Since it takes many years for a chronic *H. pylori* infection to cause gastric cancer, the required time for an *H. pylori*-dependent diffuse-type carcinogenesis should be shorter than that for the intestinal-type. A shorter latency for the diffuse-type may suggest the existence of combination of genomic alterations in the host favoring carcinogenesis, and/or distinct virulence of *H. pylori* strains (including eradication-resistant strains) behind the diffuse GAC. Therefore, the *H. pylori* treatment usually prescribed to adult patients may not be fully beneficial to prevent diffuse-type tumors, since the required molecular steps involved in this type of cancer may have already occurred before bacteria eradication. Consequently, as the prevalence of *H. pylori* infection among children has diminished significantly [16], an accompanying decline in the burden of the diffuse-type GAC would be expected; however, this has not happened so far.

Although the intestinal-type is more frequent in men than in women (2:1), the proportion of the diffuse-type is similar between genders [3, 4]. Environmental risk factors may explain the higher incidence of the intestinal-type among men, since exposure to risk factors, including tobacco, alcohol, and dietary carcinogens, is more frequent in men. On the other hand, genetic factors are thought to be more significant in women. However, none of these hypotheses explaining gender-differences have been confirmed [3].

### Location of tumors

Intestinal-type GAC has a higher incidence in the distal stomach while diffuse-type tumors are more frequently found in proximal regions [3, 4]. The incidence of distal tumors has diminished, and that of corpus and proximal non-cardia tumors has remained stable [3–5]. The incidence of distal tumors decreased in parallel with the reduction of the intestinal-type GAC. However, as distal tumors are not exclusively from the intestinal-type, at least a modest decrease in diffuse tumors would be expected if both intestinal and diffuse-types had a similar risk factor profile.

### Molecular classification

In addition to presenting phenotypic differences, diffuse GC also have specific molecular profiles, and carcinogenesis features, that differentiate it from the intestinal type. Looking at the molecular profiles of diffuse and intestinal types, there are important differences that strengthen the hypothesis that there are two diverse tumors [17–21].

The molecular characterization performed by “The Cancer Genome Atlas” Research Network (TCGA) divided the GC into four subtypes: i) Epstein-Barr virus positive tumors, presenting recurrent PIK3CA mutations, extreme DNA hypermethylation and amplification of JAK2, PD-L1 and PD-L2; ii) microsatellite unstable tumors, characterized by elevated mutational rates; iii) genomically stable tumors, enriched for the diffuse-type and iv) tumors with chromosomal instability, the most common in the intestinal-type, showing marked aneuploidy and focal amplification of receptor tyrosine kinases [17].

Analyses using online database available differentially expressed genes have been identified between these two types. Intestinal-type samples overexpressed ATPIF1, PRDX2, PRKAR2A, and SMC1A, while diffuse-type samples overexpressed DTNA, GPR161, IDS, RHOQ, and TSHZ2. These two groups of genes demonstrated distinct prognoses [22].

There are plenty of data showing differences between intestinal and diffuse molecular features [18–20] including new experimental models, as organoid tumors, presenting typical molecular profiles according to the primary tumors from where they were developed [21]. An IARC publication deeply described molecular differences among intestinal and diffuse type gastric cancer, including the sequential molecular steps supposed to be needed for each cancer type to develop [23]. Additionally, both the Asian molecular classification [24], and TCGA classification [17] highlighted the relationship among the new molecular subtypes and Lauren’s classification. Accordingly, intestinal and diffuse GAC present peculiar molecular profiles, suggesting the existence of different carcinogenic pathways distinguishing each cancer type.

### *H. pylori* infection and gastric acidity

The relationship of gastric acidity and *H. pylori* infection is well known, since this bacterium can survive, and infect gastric mucosa, mainly in distal stomach, and cause peptic diseases, in a very acid environment. Nevertheless, regarding GAC, the supposed mechanism for *H. pylori* related carcinogenesis involves stomach corpus atrophy, instead of antrum infection, and inversely, results in reduction acidity.

According to the Correa hypothesis, gastric corpus atrophy following *H. pylori* infection, which results in increasing stomach pH, may evolve to intestinal metaplasia, dysplasia and GAC, and might, partially, explain the intestinal type GAC [25]. Diffuse type carcinogenesis does not seem to be covered by this hypothesis, although there are reports relating *H. pylori* to both types of GAC.

**Fig. 1:**
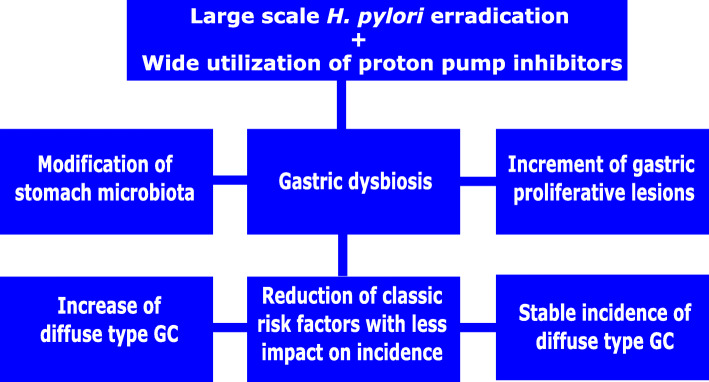
Hypothesis to explain the continuing stable incidence of diffuse-type gastric cancer

Additionally to gastric atrophy, the huge utilization of medicines [26] that results in reduction of stomach acid secretion might contribute to generate a microenvironment known to be related to GAC carcinogenesis: gastric acidity reduction. The role of such medicines in contributing to an environment prone to GAC development remains under debate, since prolonged medical inhibition of acid secretion, per se, might not be enough to trigger GAC carcinogenesis (Fig. 1). Mainly, the eventual contribution of these medicines to diffuse type GAC remains insufficiently explored. Table 1 presents comparatively the characteristics of the histological types of the Lauren.

**Table 1:** Differences between Lauren’s intestinal and diffuse types

	Lauren’s Types Gastric Adenocarcinoma	
Characteristic	Intestinal	Diffuse
Incidence trend	declining	stable or increasing
Prevalence in endemic areas	higher	lower
Environmental influence	strong	weak
Age of occurrence	elderly	young
Genetic factors	weak	strong
Male/female ratio	2:1	1:1
Location of tumor	distal	proximal
Macroscopic growth pattern	growth into the lumen	tumour spreads along the gastric wall
Carcinogenesis	well known	unknown
TCGA subtypes	mainly chromosomal instability (CIN)	mainly genomically stable (GS)
Prognosis	better	worse

### The gastric microbiota

Historically the stomach was thought to be a hostile sterile cavity, where microorganisms could not survive. *H. pylori* infection was first described in the 1980s. Recently, mainly due to new sequencing technologies, it was possible to uncover a wide variety of microorganisms in the human stomach [27].

As demonstrated for other tumor types, a gastric dysbiosis appears to take place during gastric carcinogenesis [27] and this diversity of microorganisms may differ between tumor subtypes, pointing to new causal agents. Gastric microbiota might potentially play a role in chemotherapy response, as shown to other tumors, and bacterial biotypes might serve as surrogate markers of a pre-neoplastic environment.

Whereas microbiome differences between intestinal- and diffuse-types have not been largely explored, it is possible that differences exist, and could be considered for clinical intervention. Nevertheless, the importance of gastric microbiota on cancer incidence and even on benign diseases remains uncertain [27].

Treatment of *H. pylori* infection is based on combined antibiotic therapy, associated with proton-pump inhibitors; the use of this class of drugs appears to impact the gastric microbiota [28]. Several consequences of this treatment could be hypothesized: i) reduction of non-*H. pylori* bacteria sensitive to the antibiotics; ii) increased proliferation of bacteria resistant to treatment; iii) a replacement of the microbiota due to empty niches previously occupied by antibiotic-sensitive bacteria and, ultimately, iv) a dysbiotic stomach microbiota [27, 28].

Preliminary data seem to demonstrate that in the presence of *H. pylori* infection, the diversity of other bacteria is lower than in its absence [27]. The impact of these important alterations in GC, including the diffuse-type, is unknown. Although *H. pylori* infection appears to have a key role in gastric cancer, interactions with other bacteria may be part of the carcinogenic process [27], and the understanding of these interactions might shed light on the epidemiologic trends of the diffuse-type GAC.

Gastric *H. pylori* infection is recognized as the main cause of both gastric cancer [14] and duodenal peptic ulcer. Although the etiologic agent is the same, peptic ulcer occurs in a very acid environment, and the antrum seems to be the most important site of infection [25]. On the other hand, patients with duodenal peptic ulcer have a lower risk of GAC than that of the general population [14].

**Fig. 2:**
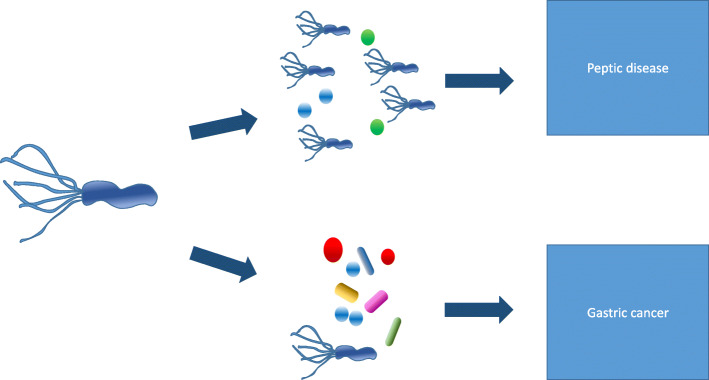
The microbiota favors the evolution to either peptic disease or gastric cancer

Interactions among different bacteria and *H. pylori* infection may influence certain molecular pathways (Fig. 2). According to hypothetical scenarios: i) *H. pylori* infection plus specific bacterial co-infection may favor gastric cancer pathway; ii) *H. pylori* infection plus other specific bacteria may favor the benign peptic disease pathway; iii) treatment of *H. pylori* infection may favor the proliferation of specific bacteria that may have a role in diffuse-type carcinogenesis, thus counteracting the *H. pylori* eradication and keeping elevated the rates of diffuse-type cancers related to stomach infection.

### Genetic predisposition

A genome-wide association study (GWAS) performed in a Japanese population identified a strong correlation between a particular single nucleotide polymorphism (SNP) rs2294008 (Met1Thr) in the Prostate Stem Cell Antigen gene (PSCA) and diffuse-type GC. The same group showed that a functional SNP in mucin 1 determines higher susceptibility to diffuse-type gastric cancer [29]. Nevertheless, among Caucasians, an association between variations in the promoter region of PSCA and GC risk has been also reported. However, for this population, the SNP rs2294008 variant carried a similar risk for diffuse and intestinal GAC types [30].

### Interfering on diffuse-type cancer burden

Several potential strategies to control gastric cancer burden have been suggested, of which the following are the most commonly adopted: i) treatment of *H. pylori* infection [12, 13]; ii) avoidance of salt-preserved food [9]; and iii) surveillance of patients at risk (gastric cancer relatives, patients with intestinal metaplasia, and hereditary cancer syndromes) [13]. However, except for the diffuse hereditary gastric cancer syndrome, which occurs in very few patients and is mainly associated with *CDH1* mutations, preventive strategies have not been effective in reducing the incidence of diffuse-type tumors.

Unlike metaplasia of the intestinal-type GAC, there are no pre-neoplastic lesions described for diffuse-type GAC that would allow surveillance or trigger preventive measures. As a consequence, innovative strategies are needed to tackle diffuse-type GAC prevention.

Looking outside the “cancer box”, an interesting phenomenon in the stomach mucosa is the increasing incidence of hyperplastic lesions [31]. Notwithstanding its benign nature, hyperplastic lesions entail high risk DNA alterations that, associated with exposure to new carcinogenic agents and to microenvironment modifications of the stomach’s mucosa, might favor a new pathway for diffuse GAC development.

Similarly to what happens regarding metaplasia and intestinal-type cancer risk, the vast majority of hyperplastic lesions will never develop as a diffuse-type cancer. Efforts to identify molecular aberrations present both in hyperplastic and other types of mucosa alterations should be investigated, in order to understand its role in diffuse-type gastric cancer.

Despite not being a consensus in current clinical practice, the identification of molecular biomarkers implicated in diffuse gastric cancer risk must be attempted. The main reason for optimism is the knowledge that molecular alterations occur many years (usually > 20 years) before cancer occurrence and that significant technology improvements seen in recent years bode well for scientific breakthroughs in this field.

In this sense it is strongly recommended the implementation of investigations addressing the role of the gastric microbiota role in gastric cancer carcinogenesis the continuous monitoring of epidemiologic trends, including differences between intestinal and diffuse GAC types.

The possible connections among *H. pylori* infection, use of antibiotics, proton pump inhibitors, and other medications, including nonsteroidal anti-inflammatory drugs, that also modify the gastric microenvironment by interfering in gastric mucus barrier, should be carefully vetted. It also deserves attention the emergence of stomach lesions such as hyperplasic polyps, and also neuro-endocrine gastric tumors, as well as the more recent diet modifications, as all these may fill central knowledge gaps, and contribute to understanding of carcinogenesis process involved in causing diffuse gastric cancer.

## Conclusion

Diffuse-type GAC differs from the intestinal-type and has unexplained and underexplored characteristics. Among these are the decline in intestinal-type incidence not accompanied by a similar decline of diffuse cancers; the fact that the impact of reducing *H. pylori* gastric infection does not seem to be as effective for the diffuse as for intestinal-type; the widespread use of proton pump inhibitors and antibiotics, which may affect the diffuse-type carcinogenic pathways; the recent increase in benign proliferative stomach lesions that could favor diffuse-type carcinogenesis; and, of great importance, modifications in gastric microbiota and the microenvironment. The knowledge of the role of these events may allow the development of innovative strategies to control gastric cancer burden.

In order to reduce the incidence of diffuse gastric cancer, it will be necessary to increase the knowledge of the carcinogenesis of this histological type, enabling the discovery of etiological factors to be fought and the recognition of early stages susceptible to effective detection and treatment.

## Data Availability

not applicable.

## Abbreviations

GAC: Gastric adenocarcinoma
GC: Gastric cancer
*H. pylori*: *Helicobacter pylori*
TCGA: The Cancer Genome Atlas
PIK3CA: Phosphatidylinositol-4,5-biphosphate 3-kinase catalytic subunit alpha
DNA: Deoxyribonucleic acid
JAK2: Janus kinase 2
PD-L1: Programmed death-ligand 1
PD-L2: Programmed death-ligand 2
ATPIF1: ATPase inhibitory factor 1
ATP: Adenosine triphosphate
*PRDX2*: Peroxiredoxin 2 gene
*PRKAR2A*: Protein kinase CAMP-dependent type II regulatory subunit alpha gene
*SMC1A*: Structural maintenance of chromosomes 1A gene
*DTNA*: Dystrobrevin alpha gene
*GPR161*: G protein-coupled receptor 161 gene
*IDS*: Iduronate 2-sulfatase gene
*RHOQ*: Ras homolog family member Q gene
*TSHZ2*: Teashirt zinc finger homeobox 2 gene
IARC: International Agency for Research on Cancer
GWAS: Genome-wide association study
SNP: Single nucleotide polymorphism
PSCA: Prostate stem cell antigen gene
*CDH1*: Cadherin-1 gene
